# Thermal Behavior of the Nimesulide-Salicylic Acid Eutectic Mixtures Prepared by Mechanosynthesis and Recrystallization

**DOI:** 10.3390/ma14247715

**Published:** 2021-12-14

**Authors:** Mona Georgescu, Viorica Meltzer, Ioana Stănculescu, Elena Pincu

**Affiliations:** Department of Physical Chemistry, Faculty of Chemistry, University of Bucharest, Regina Elisabeta Boulevard No. 4-12, 030018 Bucharest, Romania; mona.geo98@gmail.com (M.G.); viomel@gw-chimie.math.unibuc.ro (V.M.); istanculescu@nipne.ro (I.S.)

**Keywords:** nimesulide, salicylic acid, DSC, FTIR, eutectic

## Abstract

Nimesulide, salicylic acid and their binary mixtures were studied by differential scanning calorimetry (DSC) and Fourier Transform Infrared spectroscopy (FTIR). The study of such systems is a promising and viable approach for solving the problem of poor solubility of materials in general and drug systems in particular. All areas of human activity are inextricably linked to materials, and thus, the study presented in the paper and not reported in the literature is very important and provides useful data for those working in various fields. The eutectic mixtures were obtained by mechanosynthesis and by recrystallization from ethanol over the entire 0–1 range of molar fractions. For both situations at the molar fraction of nimesulide 0.5, the mixture has a eutectic that suggests an increase in solubility at this composition. The interactions that take place between the components were determined with the help of the excess thermodynamic functions (G^E^, S^E^, µ^E^), which highlight the deviation from the ideality of the considered binary systems.

## 1. Introduction

Eutectics and cocrystals are a class of new materials with physical properties other than those of singular materials such as: solubility, stability, melting temperature, melting enthalpy, dissolution rate, etc. [1,2]. Due to their improved physical properties, they have applications in many fields such as agrochemicals [3], pigments [4] and in pharmaceuticals [5]. Eutectics are solid materials composed of two or more substances that have a single solid phase at a certain temperature and composition [6]. Eutectics and cocrystals play an important role in the pharmaceutical industry due to their thermodynamic properties, which generally lead to increased solubility of poorly soluble substances [7]. The formation of eutectics is favored by certain factors, such as: (i) the compounds are immiscible in the solid state but mix after liquefaction; (ii) intimate physical interactions between the compounds that form the eutectic leading to a decrease in melting point and (iii) the tendency to interact through noncovalent interactions such as hydrogen bonding, van der Waals forces or π–π stacking interactions [8,9]. Obtaining eutectics and co-crystals is a challenge for the pharmaceutical industry. Mechanosynthesis is an advantageous method due to the fact that it does not use solvents and the process is mechanically activated. The mechanical energy required for the process is obtained by grinding [10].

**Figure 1:**
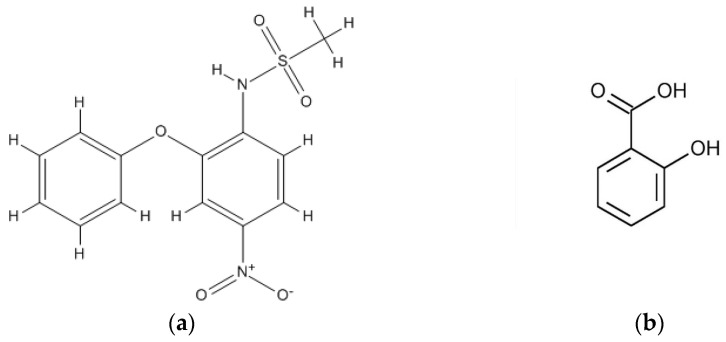
Chemical structure of: (**a**) nimesulide; (**b**) salicylic acid.

Nimesulide (NM), N-(4-Nitro-2-phenoxyphenyl)methanesulfonamide is a non-steroidal anti-inflammatory drug (NSAIDs), which has three major therapeutic actions, reducing: inflammation (anti-inflammatory effect), pain (analgesic effect) and fever (antipyretic effect). Nimesulide differs from other nonsteroidal anti-inflammatory drugs in that it contains a weakly acidic sulfonanilide group (Figure 1a) instead of a carboxylic one [11]. The Biopharmaceutics Classification System (BCS) groups nimesulide in class II, which means that it has low solubility and high permeability [12]. Nimesulide is a potent and selective inhibitor of cyclooxygenase-2, very effective in the treatment of various forms of pain and inflammatory conditions with minimal side effects and low toxicity [13,14] and is a potential drug for future treatment strategies against the SARS-CoV-2 virus, as it appears to completely eliminate, indirectly, the B0AT1 protein transport function [15]. Two polymorphic forms of nimesulide have been reported in the literature: form I [16] and form II [17,18]. In order to have good bioavailability, the drugs must be soluble in the gastric juice, in the case of oral administration or in appropriate solvents to improve transdermal permeability [19]. The administration of nimesulide through the skin could offer many benefits compared to oral administration by avoiding metabolism in the liver, as well as gastrointestinal side effects. Adverse reactions such as nausea, vomiting, diarrhea and elevated serum liver enzymes have been reported for nimesulide [20]. Many attempts to improve the solubility and dissolution rate of nimesulide have been reported in the literature, including: eutectic formation with nicotinamide [21], solid dispersions with PEG and PVP [22,23], complexation with cyclodextrins [24].

Salicylic acid (SA) (Figure 1b) is a hydroxyacid with the molecular formula: C_7_H_6_O_3_. Salicylic acid is freely soluble in ethanol and in water has a solubility of 2.48 g/L at 25 °C. It is used externally on the skin, where it exerts a weak antiseptic action and a keratolytic action, peels and removes keratin from the skin. This property gives salicylic acid the role of a beneficial agent in the local treatment of warts, fungal infections and certain forms of eczematous dermatitis. Salicylic acid is also used as an exfoliant in cosmetic dermatology [25]. Salicylic acid does not show polymorphism and the presence of the -COOH group makes it a good coformer for obtaining eutectics or cocrystals [26,27,28].

Differential scanning calorimetry (DSC) is one of the most common analytical techniques used to characterize solids and implicitly the pharmaceutical ingredients both singular and in mixtures [29]. DSC is a very precise and accessible technique to determine the formation of cocrystals or eutectics. Many researchers have used this technique to determine the formation of eutectics and cocrystals between nimesulide and various coformers such as: nicotinamide [21], PVP [22], PEG [22,23], urea [23] and pyridine analogues [30]. To the best of our knowledge, only DSC data of pure nimesulide and, respectively, salicylic acid are found in the literature [24,25,26,27]. FTIR spectroscopy is widely used in conjunction with DSC and/or other techniques to characterize chemical structure modifications and molecular interactions in any physical state due to the high selectivity, low analysis costs and rapid sample preparation [21,22,30,31,32].

The aims of this research were: (i) to obtain a eutectic mixture or cocrystal between the two studied active principle ingredients (API), nimesulide (NM) and salicylic acid (SA) by mechanosynthesis, a solvent-free, green method and respectively by recrystallization from ethanol and (ii) to characterize the eutectic mixtures by differential scanning calorimetry and FTIR spectroscopy. The article is original, carrying out a thermal and spectral study of a binary mixture, nimesulide–salicylic acid, not reported in the literature. The presented results have a fundamental and applicative resonance, on the one hand in green chemistry and on the other hand in the realization of some new drugs with high solubility, as a consequence of the formation of a eutectic mixture.

## 2. Materials and Methods

### 2.1. Chemicals

For this study, nimesulide (purity ≥98%), salicylic acid (purity ≥98%), ethanol (purity 99%) and acetone (purity, 99%) from Merck, Darmstadt, Germany, were used without further purification.

### 2.2. Equipment

The experimental study was performed with a Perkin Elmer Diamond DSC calorimeter, in the temperature range 20–170 °C, where all processes were detected, with a heating rate of 10 K/min in an inert argon atmosphere. The heating rate of 10 K/min was used because it is the rate at which the thermal decalage between sample and reference is negligible. The heating rate modification affects by small shifts only the temperatures of the processes.

Visual analysis of the eutectic nimesulide–salicylic acid mixture was performed using Böetius Thermomicroscope equipped with an ortho-ocular 16× with a heating rate of 4 K/min.

FTIR data were taken in the 4000–400 cm^−1^ domain with a Vertex 70 spectrometer from Bruker, Ettlingen, Germany, with the following working parameters: 4 cm^−1^ resolution, 0.1 cm^−1^ wavenumber accuracy, 0.1% T photometric accuracy and 64 scans. A KBr beam splitter and an RTDLaTGS (Room Temperature Deuterated Lanthanum α Alanine doped Triglycine Sulphate) detector were employed. The measurements were performed by the technique of pastillation with KBr of spectroscopic purity (Merck reagent) at a reference:sample weight ratio of 300:1 toward a 300 mg KBr reference disc. The discs were prepared by pressing at 10 t/cm^2^, under vacuum, the fine powder obtained after grinding the samples for 5 min in an agate mortar. Spectra were processed with the OPUS software stepwise using: atmospheric compensation for CO_2_ and H_2_O signals elimination, vector normalization, baseline correction with straight lines and one iteration additional concave rubber band correction and automatic peak peaking.

### 2.3. Preparation of Binary Mixtures

The binary mixtures were prepared in two ways: by mechanosynthesis (physical mixture) and by recrystallization from ethanol. The binary mixtures were prepared stepwise, with an 0.1 molar fraction increment, over the entire range of molar fractions, between 0 and 1.

#### 2.3.1. Physical Mixture Preparation

After weighing the two components, they were mixed using a mortar and pestle for 10 min at room temperature (298.15 K) until a fine powder was obtained, which was then analyzed by DSC and FTIR, respectively.

#### 2.3.2. Recrystallization by Slow Evaporation Method

The recrystallization was made from ethanol solution of different molar fractions in the 0–1 molar fraction interval. The desired amount of nimesulide and salicylic acid was weighted, and 100 mL ethanol was added. The solution was mixed in a beaker on a magnetic stirrer with 100 rpm for 60 min. The obtained solution was transferred to a Petri dish covered with a perforated aluminum foil and left to evaporate at room temperature until a dry mixture was obtained and the resulting remnant was analyzed.

### 2.4. Data Analysis

The activity coefficients of the two components as a function of temperature and composition were calculated with the following formula [33]:

(1) $$-{\ln\gamma }_{i}^{l}=\frac{\Delta^{t}H_{i}^{0}}{R}\left[\frac{1}{T}-\frac{1}{T_{i}^{0}}\right]+{\mathrm{lnx}}_{i}^{l}\;,$$

where:$\;\Delta^{t}H_{1}^{0}$ = molar melting enthalpy of component i of the system;

$\gamma_{i}$ = the activity coefficient of component i;

$x_{i}=$ the molar fraction for component i;

R = universal gas constant;

T = melting temperature of the binary system;

$T_{i}^{0}$ = melting temperature of component i.

In order to obtain more information on the nature of the interactions that take place in the real system, the values of the excess thermodynamic functions were calculated with the following equations [34]:

(2) $$\mu_{i}^{E}=\;\mathrm{RT}\cdot \ln\gamma_{i}$$

(3) $$G^{E}=\mathrm{RT}\left[x_{1}\ln\gamma_{1}^{l}+x_{2}\ln\gamma_{2}^{l}\right]$$

(4) $$S^{E}=-R\left[x_{1}\ln\gamma_{1}^{l}+x_{2}\ln\gamma_{2}^{l}\right]-\mathrm{RT}\left[x_{1}\frac{\partial \ln\gamma_{1}^{l}}{\partial T}+x_{2}\frac{\partial \ln\gamma_{2}^{l}}{\partial T}\right]$$

In the case of the binary physical mixture nimesulide–salicylic acid, the structure of the eutectic was determined by calculating the roughness factor (α). It was calculated using the relation [35,36]:

(5) $$\alpha =\xi \frac{\Delta H}{\mathrm{RT}}=\xi \frac{\Delta S}{R},\;$$

where ΔH represents the melting enthalpy, and ξ is a crystallographic factor, which depends on the geometry of the molecules, has a value less than or equal to 1 and represents the fraction of the total number of neighbors located in the new formed layer.

The melting enthalpy for the nimesulide–salicylic acid mixture was calculated using the equation:

(6) $$\Delta^{M}H={\left(\Delta^{t}H\right)}_{\exp}-{\left(\Delta^{t}H\right)}_{\mathrm{calc}}\;,$$

where ${\left(\Delta^{t}H\right)}_{\exp}$ represents the melting enthalpy determined experimentally from DSC data. The term ${\left(\Delta^{t}H\right)}_{\mathrm{calc}}$ represents the melting enthalpy for a binary system, calculated additively with the mixing rule [37,38]:

(7) $${\left(\Delta^{t}H\right)}_{\mathrm{calc}}=x_{1}\Delta^{t}H_{1}^{0}+x_{2}\Delta^{t}H_{2}^{0}\;,$$

where: $x_{i}$ represents the molar fraction of component i, and $\Delta^{t}H_{i}^{0}$ is the molar melting enthalpy for component i.

## 3. Results and Discussion

### 3.1. DSC Study of Nimesulide–Salicylic Acid Physical Mixtures

**Figure 2:**
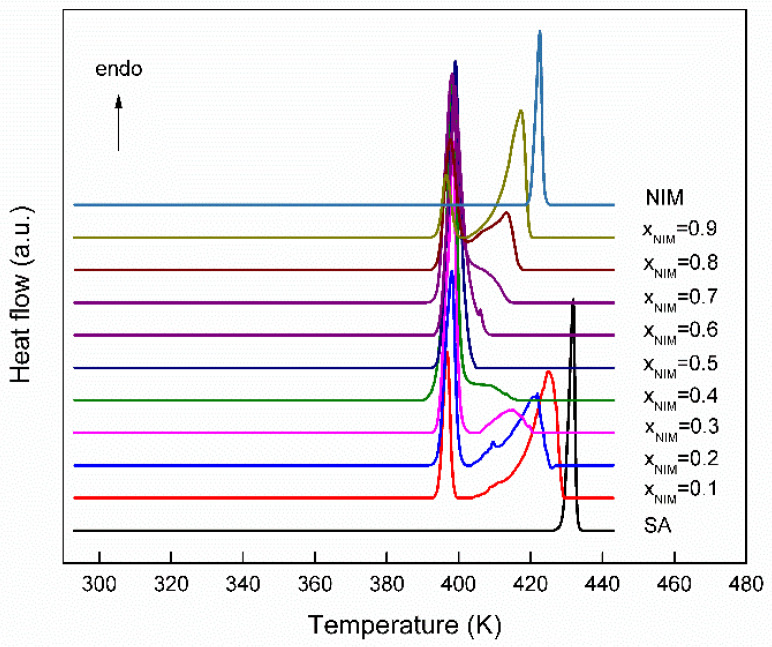
The DSC curves for the nimesulide–salicylic acid physical mixtures at first heating.

The initial study was performed on pure commercial active principles ingredients as received and recrystallized from ethanol, respectively. From the DSC data obtained for the nimesulide–salicylic acid physical mixture on first heating (Figure 2), it can be seen that all the compositions studied show an endothermic process at approximately the same temperature of 394 K (value that is lower than the temperatures of the pure components), which reveals that the system is characterized by a eutectic point. For molar fractions smaller and larger than 0.5, the second endothermic process characteristic of the component that is in excess of the eutectic composition may be observed. The eutectic reaction is confirmed by the first peak, which consistently appeared at the same temperature, 394 K, for all compositions, which means the new solid phase is formed. In the literature, the eutectic reaction for nimesulide–nicotinamide [21] binary mixture at a temperature of 383 K was obtained, and in this case, the solubility of eutectic was higher than that of the nimesulide parent drug.

From the DSC curve obtained for pure nimesulide, it can be seen that it melts without decomposition. At the first heating, a single endothermic process appears at 419.67 ± 0.40 K, which is attributed to the melting of the polymorphic form I and characterized by an enthalpy of melting equal to 99.42 J/g (30.65 ± 0.27 kJ/mol). The value of the temperature at which the endothermic process is found corresponds to the existing data in the literature [24,39,40,41].

The thermal study of salicylic acid reveals that in the temperature range between 293.15 K and 443.15 K, appears a single endothermic process at a temperature of 429.70 ± 0.20 K, attributed to the first-order solid–liquid phase transition. The value of the temperature at which the endothermic process is observed corresponds to the literature data [42,43].

The thermodynamic parameters obtained from the thermal study of the nimesulide–salicylic acid binary physical mixtures at the first heating are presented in Table 1.

**Figure 3:**
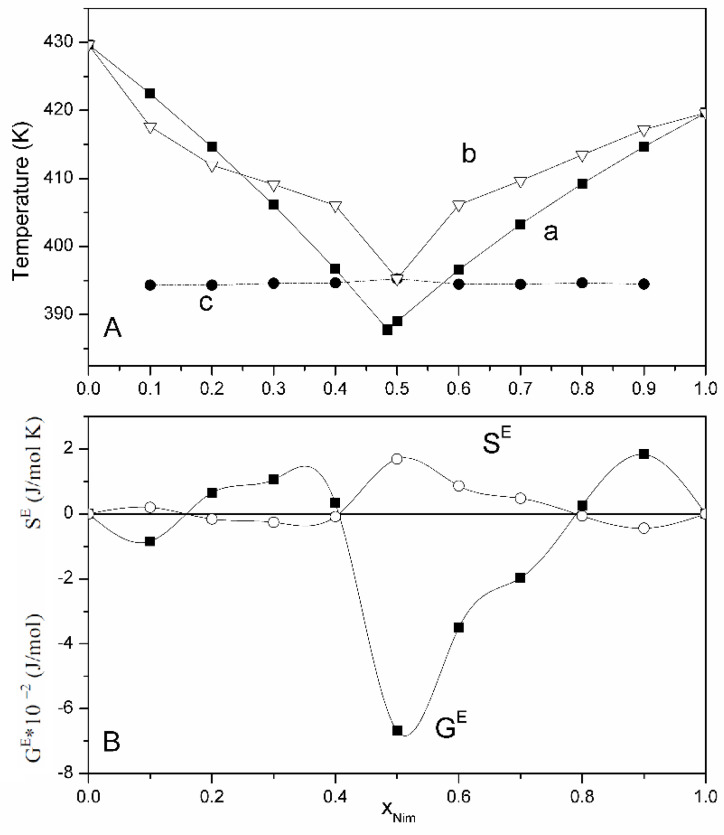
(**A**). Phase diagram of binary physical mixture nimesulide–salicylic acid: (a) calculated under ideal conditions; (b) determined experimentally; (c) for the eutectic composition. (**B**). Graphical representation of excess functions (excess Gibbs free energy and entropy).

The phase diagram obtained for the ideal system is compared with that of the real binary system for observing the deviation from the ideality (Figure 3A). In order to obtain more information on the nature of the interactions that take place in the real system, the values of the excess thermodynamic functions (that are shown in Table 2) were calculated, and their graphical representation according to the molar fraction of nimesulide is shown in Figure 3B. For the molar fractions of nimesulide between 0–0.1634 and 0.4–0.8, the excess Gibbs free energy is less than 0, and the entropy is greater than 0, which means that attractive forces between different molecules develop. For the range of nimesulide molar fractions between 0.1634–0.4 and 0.8–1, the excess Gibbs free energy is greater than 0 and the entropy is less than 0 due to the affinity between the same molecules.

**Figure 4:**
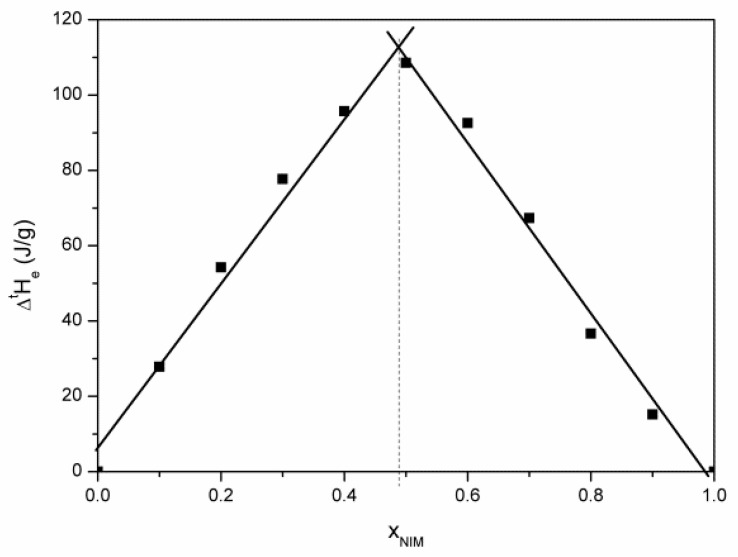
Tammann diagram for nimesulide–salicylic acid physical mixture.

From melting enthalpies of the eutectic composition, we constructed the Tammann diagram (Figure 4), which helps to determine the eutectic composition with great precision. The maximum value of the melting enthalpy of the eutectic composition is found at the value of the molar fraction of nimesulide equal to 0.4885.

The exact determination of the eutectic point is very important because any excess of a component can lead to the modification of the physical properties of the mixture, including the solubility [6]. For the binary physical mixture nimesulide (drug)–salicylic acid (coformer), we obtained a eutectic with the molar ratio of 1:1, which has the lowest Gibbs free energy (negative), which means that a heterosynthon between the nimesulide and salicylic acid molecules was formed [10].

**Figure 5:**
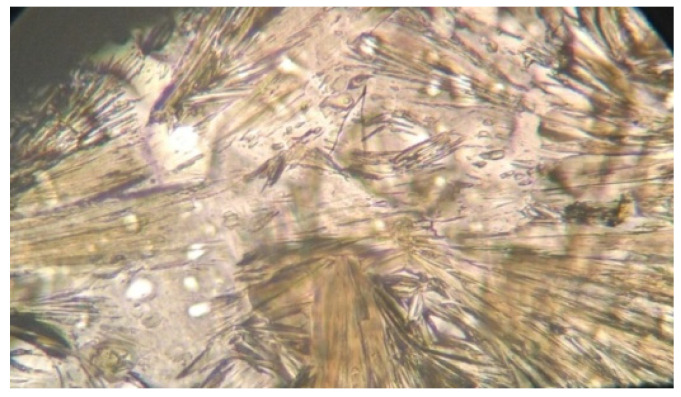
Descriptive visual analysis of the NM-SA eutectic microstructure at 16× magnification obtained during cooling with a thermomicroscope.

The value of the roughness factor of the eutectic composition is 7.60 and reveals that it has an irregular structure, which increases on crystalline facets, a fact illustrated by the analysis of the binary physical mixture nimesulide–salicylic acid at the Böetius thermomicroscope (Figure 5). The solid–liquid interface is smooth and advances into the liquid by propagating atoms at the interface.

The value of the mixing enthalpy of eutectic was also determined, and it is equal to −1.23 kJ/mol. The value of the mixing enthalpy being less than 0, the structure of the eutectic is of cluster type [44].

### 3.2. DSC Study of Nimesulide–Salicilic Acid Binary Mixtures Recrystalized from Ethanol

**Figure 6:**
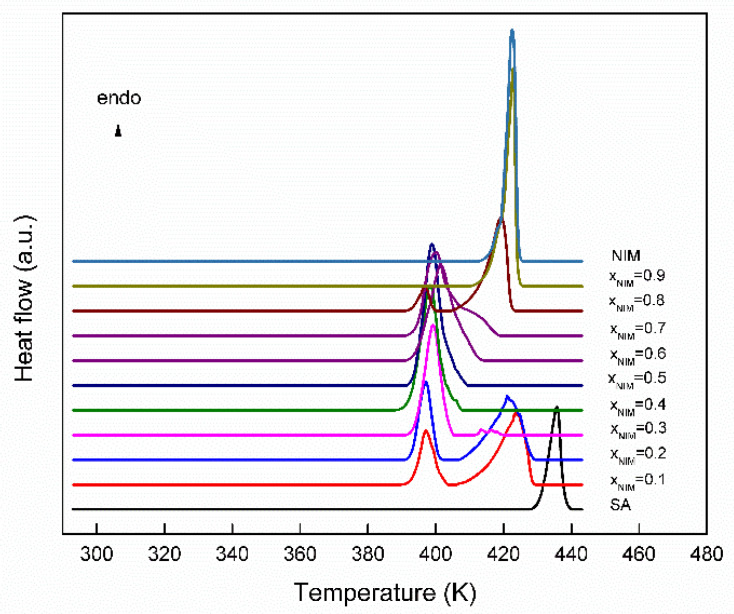
The DSC curves for the nimesulide–salicylic acid binary system recrystallized from ethanol at first heating.

From the DSC data obtained for the nimesulide–salicylic acid binary mixture recrystallized from ethanol on first heating (Figure 6), it can be seen that all the compositions studied show an endothermic process around 394 K (value that is lower than the temperatures of the pure components), which reveals that the system is characterized by a eutectic mixture. An exception to the formation of the eutectic mixture is the nimesulide–salicylic acid binary mixture recrystallized from ethanol, for which the molar fraction of nimesulide is 0.9. For molar fractions smaller and larger than 0.5, the second endothermic process characteristic of the component that is in excess may be observed.

The thermodynamic parameters obtained from the thermal study of the binary mixtures nimesulide–salicylic acid recrystallized from ethanol at the first heating are presented in Table 3.

**Figure 7:**
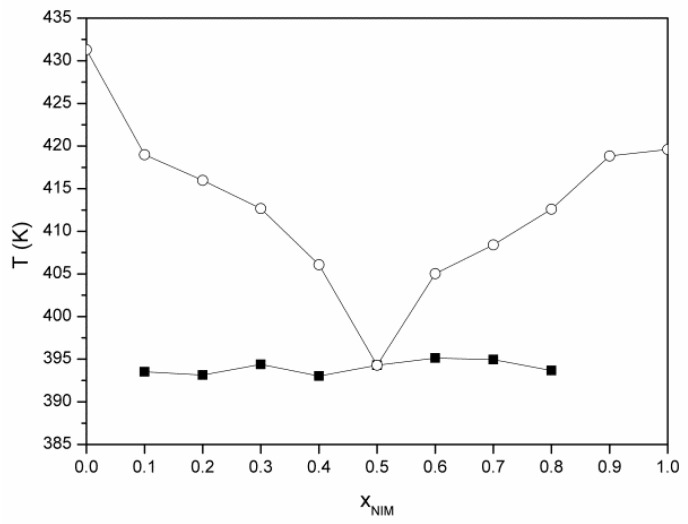
Phase diagram for nimesulide–salicylic acid binary mixtures recrystallized from ethanol.

**Figure 8:**
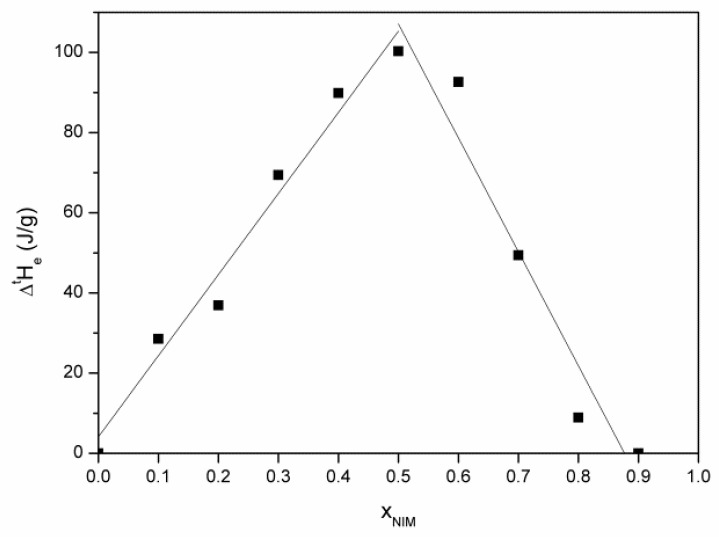
Tammann diagram corresponding to the nimesulide–salicylic acid binary mixture recrystallized from ethanol.

The phase diagram for the nimesulide–salicylic acid binary mixtures recrystallized from ethanol (Figure 7) can be constructed by using the melting temperatures from the DSC curves for the eutectic composition and for the excess compositions. The Tammann diagram (Figure 8) starts at approximately the value of the molar fraction of nimesulide equal to 0 (x_NIM_ = 0) but ends at the value of the molar fraction of nimesulide equal to 0.8826 (x_NIM_ = 0.8826), which underlines that the two components of the mixture are miscible in solid phase for this molar fraction. The maximum value of the melting enthalpy of the eutectic composition is found for the value of the molar fraction of nimesulide equal to 0.5025.

The composition of eutectic for mixtures recrystallized from ethanol is similar to that of the mixture obtained by mechanosynthesis and has a melting enthalpy very close to it, which means that the two systems have similar physical properties. Enhancement of dissolution rate and solubility of nimesulide is expected as shown in our previous work on bromazepam–citric acid eutectic mixture [7] and also in a similar study of nimesulide–nicotinamide eutectic composition [21].

### 3.3. Analysis by FTIR Spectrometry

**Figure 9:**
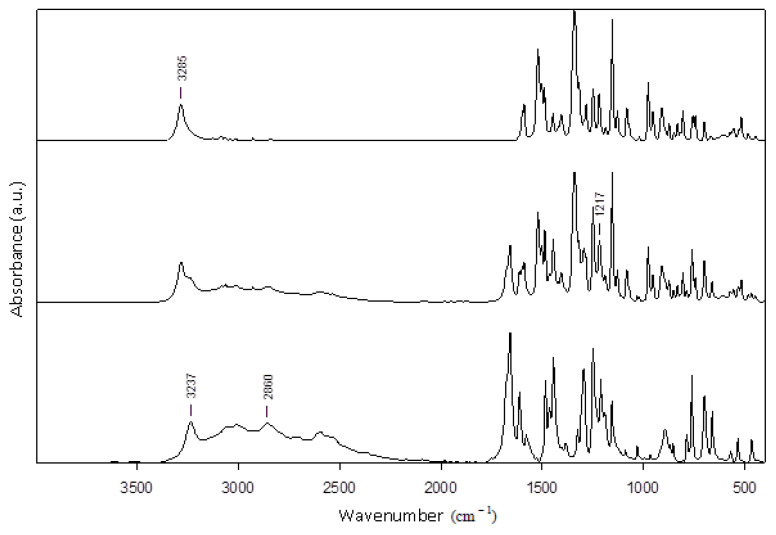
Graphical representation of FTIR spectra for: pure nimesulide (top line), physical mixture with x_NIM_ = 0.5 (middle line), and pure salicylic acid (bottom line).

**Figure 10:**
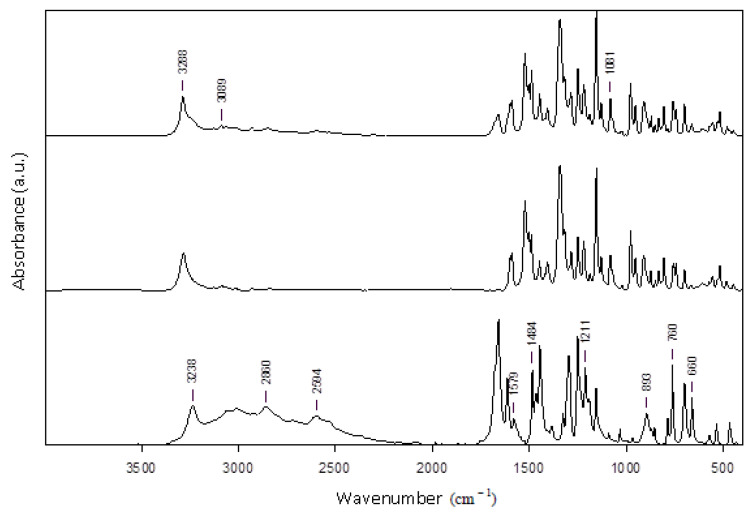
Graphical representation of FTIR spectra for: binary mixture recrystallized from ethanol with x_NIM_ = 0.5 (top line), nimesulide recrystallized from ethanol (middle line), and salicylic acid recrystallized from ethanol (bottom line).

The nimesulide–salicylic acid binary physical mixture with x_NIM_ = 0.5 (Figure 9) and the nimesulide–salicylic acid binary mixture recrystallized from ethanol with x_NIM_ = 0.5 (Figure 10) were analyzed by FTIR spectrometry.

The FTIR spectra of pure nimesulide reveal its main modes of vibration at: 1589 cm^−1^, due to C=C of aromatic rings; 1154 cm^−1^, due to the sulfonyl –SO_2_ group; 1521 cm^−1^, due to the nitro –NO_2_ functional group; 1249 cm^−1^, due to the simple C–O diaryl bond and at 3285 cm^−1^ due to the N–H bond of the sulfonamide group. Additionally, the characteristic band of the vibration of the S–N bond in the sulfonamide group at 952 cm^−1^ is noticeable.

The characteristic bands of pure salicylic acid functional groups are observed in the FTIR spectra: hydroxyl group, –OH of –COOH, at 3237 cm^−1^; C–H bond at 2861 cm^−1^; carbonyl group, C=O of –COOH, at 1658 cm^−1^; C=C double bonds of the aromatic ring at 1612 cm^−1^ and 1578 cm^−1^; phenolic hydroxyl group, –OH of the aromatic ring, at 1325 cm^−1^; simple C–O bond of –COOH at 1295 cm^−1^; unsaturated =C–H at 760 cm^−1^ and 698 cm^−1^. Additionally, the FTIR spectra of pure salicylic acid reveal some peaks in the range 1444–1484 cm^−1^, attributed to the characteristic vibration of the C–C simple bond, and in the range 1190–1249 cm^−1^, attributed to the characteristic vibration of the phenolic C–OH bond.

Comparison of the spectra of pure, as-received API’s powders and of recrystallized-from-ethanol APIs reveals only very small shifts of the FTIR peaks within the wavenumber accuracy interval, of about 0.1 cm^−1^.

All of the above characteristic bands of nimesulide and salicylic acid are also found in the FTIR spectra of the physical mixture and recrystallized from ethanol mixture at approximately the same value of the wavenumber, indicating the absence of polymorphism. For both eutectics, the observed shifts of bands position indicate the presence of intermolecular interactions between the two APIs. The spectra of the mixture recrystallized from ethanol at x_NIM_ = 0.9 do not show major modifications, although after this molar ratio the two APIs are miscible in the solid phase, as shown from the Tammann diagram. The highest bands shift, of few cm^−1^, detected in the eutectic obtained by physical mixture spectra are observed for the hydroxyl, CH bending and skeletal vibrations, indicating a hydrogen-bonding network and, in general, non-specific physical interaction modification influencing the molecular structure packing in the crystal. The overall spectral modification shows the formation at a 0.5 molar ratio of similar supermolecular structure eutectics for both the physical mixture and recrystallized-from-ethanol mixture in agreement with DSC results that indicate the formation of eutectics with the same properties. As emphasized, general and specific attractive electrostatic forces develop between the drug and conformer, as may be seen from the several small band shifts highlighted in the FTIR spectra of Figure 9 and Figure 10. Thus, the hydroxyl group of pure SA at 3237 cm^−1^ is shifted to a higher wavenumber in both eutectic FTIR spectra, indicating the participation in the formation of hydrogen bonds with the NM hydrogen bond donors and acceptors groups. Most probably, a heterosynthon is formed via the -NH group of NM, as may be deduced from the shifts of this band from 3285 cm^−1^ in pure NM to 3288 cm^−1^ in the eutectic. Other bands shifts are observed for stretching the aromatic C-H vibration at 3089 cm^−1^ in the eutectic, revealing π–π interactions. Additionally, in the finger print region, below 1700 cm^−1^, other vibration bands of C-O bonds at 1211 and of C=C at 1579 cm^−1^ in the SA spectra are shifted to higher wavenumbers in the eutectic mixtures, showing participation in hydrogen bonds’ formation and to π–π stacking interactions. Several other FTIR bands due to skeletal vibrations, polar and nonpolar groups may be seen confirming the existence of intermolecular attractive interactions between the two APIs at a 0.5 molar ratio.

## 4. Conclusions

The nimesulide–salicylic acid binary physical mixtures have a simple eutectic for the entire range of molar fractions. The Tammann diagram starts at approximately x_NIM_ = 0 and ends at x_NIM_ = 1, which emphasizes that the two components of the mixture are not miscible in the solid phase. The maximum value of the melting enthalpy of the eutectic composition is found for the value of the molar fraction of nimesulide equal to 0.4885. The nimesulide–salicylic acid binary mixtures recrystallized from ethanol have a simple eutectic for the range of molar fractions of nimesulide 0.1–0.8. The Tammann diagram starts at approximately x_NIM_ = 0 and ends at x_NIM_ = 0.8826, which shows that the two components of the mixture are miscible in the solid phase for this molar fraction, and the maximum value of the melting enthalpy of the eutectic composition is found at x_NIM_ = 0.5025. FTIR spectra of physical mixtures and recrystallized mixtures from ethanol did not confirm the presence of a co-crystal, instead revealing only small differences in the bands position due to hydrogen bonds and general van der Walls interactions. For both binary mixtures, the eutectic composition is around the 0.5 molar fractions, and the eutectic temperature is approximately the same. In conclusion, the eutectic composition does not show polymorphism, and as methods of preparation of binary mixtures, both may be used successfully depending on the purpose of their use. Polymorphism in eutectic is unlikely, and thus, the eutectic preparation leads to a new solid form devoid of polymorphism in sharp contrast to co-crystals. Co-crystals may present polymorphism that must be studied to avoid obtaining inactive forms. The eutectics obtained by recrystallization and mechanosynthesis have the same molar fraction and the same temperature, which means that they have the same solid phase and crystal form. In conclusion, both preparation methods may be used because the same eutectic is obtained, which is an advantage when aiming to obtain new enhanced solubility pharmaceutical forms by solvent-free green technologies.

## Figures and Tables

**Table 1:** Thermodynamic parameters for the nimesulide–salicylic acid physical mixtures at first heating.

x_NIM_	T_e_ (K)	T (K)	Δ^t^H_e_ (J/g)
0	-	429.7	0
0.1	394.34	417.63	28.43
0.2	394.34	411.96	55.83
0.3	394.6	409.13	77.7
0.4	394.65	406.05	91.62
0.5	395.27	395.27	109.69
0.6	394.46	406.13	96.53
0.7	394.47	409.67	54.87
0.8	394.66	413.48	35.62
0.9	394.48	417.21	16.32
1	-	419.67	0

x_NIM_—molar fraction of nimesulide, T_e_—melting temperature of eutectic, T—melting temperature of component in excess, Δ^t^He—melting enthalpy of eutectic composition.

**Table 2:** Activity coefficient values and excess thermodynamic functions calculated for the binary physical mixture nimesulide–salicylic acid.

x_NIM_	x_SA_	T (K)	ln γ_NIM_	ln γ_SA_	G^E^ (J/mol)	S^E^ (J/mol٠K)	$\mu_{NIM}^{E}$(J/mol٠K)
0	1	429.7	-	0	0	0	-
0.1	0.9	417.63	0.4064	−0.0721	−84.33	0.20	1410.94
0.2	0.8	411.96	0.2604	−0.0413	65.17	−0.16	892.01
0.3	0.7	409.13	−0.0085	0.0479	105.37	−0.26	−28.91
0.4	0.6	406.05	−0.2053	0.1531	32.93	−0.08	−693.04
0.5	0.5	395.27	0.1508	−0.5577	−668.46	1.69	495.73
0.6	0.4	406.13	0.2179	−0.5862	−350.18	0.86	735.89
0.7	0.3	409.67	0.1422	−0.525	−197.32	0.48	484.45
0.8	0.2	413.48	0.0916	−0.3293	25.61	−0.06	314.98
0.9	0.1	417.21	0.0536	0.0461	183.19	−0.44	185.79
1	0	419.67	0	-	0	0	0

x_NIM_—molar fraction of nimesulide, x_SA_—molar fraction of salicylic acid, T—liquidus temperature, **γ_NIM_**—activity coefficient of nimesulide, **γ_SA_**—activity coefficient of salicylic acid, G^E^—excess Gibbs free energy, S^E^—excess entropy, $\mu_{\mathrm{NIM}}^{E}$—excess chemical potential of nimesulide.

**Table 3:** Thermodynamic parameters for the nimesulide–salicylic acid binary system recrystallized from ethanol at first heating.

x_NIM_	T_e_ (K)	T (K)	Δ^t^H_e_ (J/g)
0	-	431.29	0
0.1	393.52	418.99	31.73
0.2	393.13	415.98	36.91
0.3	394.39	412.67	69.42
0.4	393.02	406.08	89.87
0.5	394.29	394.29	100.28
0.6	395.13	405.02	92.63
0.7	394.94	408.4	49.4
0.8	393.66	412.6	8.94
0.9	-	418.84	0
1	-	419.61	0

x_NIM_—molar fraction of nimesulide, T_e_—melting temperature of eutectic, T—melting temperature, of component in excess, Δ^t^H_e_—melting enthalpy of eutectic composition.

## Data Availability

The data supporting reported results are available on request from the authors.
